# Anti-ganglioside antibodies in patients with Zika virus infection-associated Guillain-Barré Syndrome in Brazil

**DOI:** 10.1371/journal.pntd.0007695

**Published:** 2019-09-17

**Authors:** Juan Rivera-Correa, Isadora Cristina de Siqueira, Sabrina Mota, Mateus Santana do Rosário, Pedro Antônio Pereira de Jesus, Luiz Carlos Junior Alcantara, Joel D. Ernst, Ana Rodriguez

**Affiliations:** 1 New York University School of Medicine, Dept. of Microbiology, New York, New York, United States of America; 2 Instituto Gonçalo Moniz, Fundação Oswaldo Cruz, Ministério da Saúde, Salvador, Brazil; 3 Hospital Santa Izabel, Salvador, Brazil; 4 Hospital Geral Roberto Santos, Secretaria Estadual da Saúde da Bahia, Salvador, Brazil; 5 Laboratório de Flavivírus—Instituto Oswaldo Cruz—Fundação Oswaldo Cruz, Rio de Janeiro, Brazil; 6 Division of Experimental Medicine, University of California, San Francisco, San Francisco, CA, United States of America; Louisiana State University, UNITED STATES

## Abstract

Zika virus infection is associated with the development of Guillain-Barré syndrome (GBS), a neurological autoimmune disorder caused by immune recognition of gangliosides and other components at nerve membranes. Using a high-throughput ELISA, we have analyzed the anti-glycolipid antibody profile, including gangliosides, of plasma samples from patients with Zika infections associated or not with GBS in Salvador, Brazil. We have observed that Zika patients that develop GBS present higher levels of anti-ganglioside antibodies when compared to Zika patients without GBS. We also observed that a broad repertoire of gangliosides was targeted by both IgM and IgG anti-self antibodies in these patients. Since Zika virus infects neurons, which contain membrane gangliosides, antigen presentation of these infected cells may trigger the observed autoimmune anti-ganglioside antibodies suggesting direct infection-induced autoantibodies as a cause leading to GBS development. Collectively, our results establish a link between anti-ganglioside antibodies and Zika-associated GBS in patients.

## Author summary

Zika virus infection can trigger the development of Guillain Barré syndrome (GBS), a neurological autoimmune disorder mediated by antibodies recognizing gangliosides in nerve membranes. Mechanisms such as molecular mimicry have been identified as a cause for GBS development in certain infections, such as *Campylobacter jejuni*, but the broad self reactivity observed during GBS suggests a role for alternative mechanisms. Our finding that Zika patients with GBS present higher levels of anti-ganglioside antibodies compared to uncomplicated Zika patients in Brazil points to these auto-antibodies as a trigger for GBS in these patients. These findings further support infection-induced autoantibodies as a factor contributing to GBS development, adding novel mechanisms for GBS development beyond molecular mimicry.

## Introduction

Zika virus is an arbovirus (arthropod-borne) of the Flaviviridae family, which like dengue viruses and alphavirus chikungunya virus is transmitted by Aedes mosquitoes. Although discovered in 1947 in Uganda, the first large Zika virus outbreak was reported in Micronesia in 2007 [1] followed by the 2014 French Polynesia outbreak [2] and the massive Latin American outbreak in 2015, which was first reported in Brazil and spread across the Americas [3]. During this outbreak, alarming Zika-associated complications, such as microcephaly and Guillain Barré syndrome (GBS) were reported [4, 5].

GBS is an inflammatory neuropathy and the most common cause of neuromuscular paralysis in the world [6]. The etiology of GBS is unknown but its development has been highly associated with post-infection autoimmune responses against gangliosides in peripheral nerves. Gangliosides are sialated glycosphingolipids found in neuronal membranes and are involved in different neuronal functions. Autoimmune antibodies recognizing gangliosides are found in a high proportion of patients with GBS (62% [12]) and are thought to contribute to neuronal pathology inducing complement-mediated axonal injury and demyelination [6]. Molecular mimicry has been proposed as a likely mechanism in infection-induced GBS, where antibodies generated against microbial antigens with structural similarities to specific gangliosides would cross-react with host gangliosides in neuronal membranes. A classic example is GBS associated with *Campylobacter jejuni* infection [7]. Zika virus was added to the list of GBS-associated pathogens due to the high incidence reported during the 2015 Latin America outbreak [8]; however, Zika virus-associated GBS shows anti-gangliosides antibodies (anti-GA1) that cannot be attributed to molecular mimicry [9], as described for *C*. *jejuni* [7], suggesting alternative mechanisms for the generation of autoantibodies as a result of Zika infection.

During many autoimmune disorders, such as rheumatoid arthritis, autoantibodies play an essential pathological role in mediating the disease. Interestingly, increased levels of IgG autoantibodies against the ganglioside GD3 have been observed in patients with acute Zika infection and without neurologic manifestations such as GBS [10]. Some GBS manifestations have also been associated with elevated levels of autoantibodies such as anti-ganglioside antibodies that can target peripheral nerves [11, 12], but the association of these antibodies with Zika-induced GBS remains unclear.

In this study we evaluate the antibody reactivity levels against 17 different glycolipids, including mostly gangliosides, presented in single and combination form, in the plasma of Zika-infected patients from one of the locations of the 2015 outbreak in Salvador, Brazil. We observed that Zika-associated GBS patients have significantly higher levels of plasma anti-glycolipid antibodies compared to non-GBS Zika-infected patients. We also observed a broad repertoire of glycolipids, including gangliosides, that were targeted by both IgM and IgG anti-self antibodies. Collectively, these results established a link between anti-ganglioside antibodies and Zika-associated GBS patients.

## Methods

### Ethics statement

This study was approved by the institutional review board of Instituto Gonçalo Moniz-Fiocruz–n°1184454/2015. All participants were adults, agreed to participate in the study and signed Informed Consent.

### Study design and sample collection

Cases of GBS and encephalitis associated with arbovirus infection and Zika infection without neurological symptoms were enrolled in a surveillance study in neurological units of two reference hospitals in Salvador, Bahia, Brazil, from May 2015 to April 2016, during the Zika outbreak in this area [13]. The study population were patients with acute neurological syndromes admitted to neurology sectors of participating hospitals. Patients with Zika infections but no neurological signs were recruited as part of a surveillance program for Zika infections in the same hospitals. All patients with neurological syndromes were evaluated by the researcher neurologist and the diagnosis of GBS was established according to international criteria [14]. The inclusion criteria were: (1) Patients with symptoms compatible with GBS and its variants or encephalitis. The diagnosis of GBS, Miller-Fisher syndrome (MFS) and its variants [14]; and encephalitis [15] was predetermined by disease-specific criteria. [2] Patients that reported acute exantemathous or fever illness in the 4 weeks before onset of neurologic symptoms. Electromyography and nerve conduction studies were performed in patients with GBS. See Table 1 for details regarding the timing of neurologic symptoms and sample collection in relations to symptoms of arbovirus infection.

**Table 1:** Patient diagnosis and detection of Zika RNA (by RT-PCR) and arbovirus IgM and IgG by ELISA.

	Patient ID	Zika				GBS	Time to neuro onset (days)^1^	Time to sample collection (days)^2^	Chikungunya			Dengue		
		RNA	NT^3^	IgM	IgG	Type			RNA	IgM	IgG	RNA	IgM	IgG
Acute Zika (n = 3)	1	+		+	+	-		2	-	-	-	-	-	+
	2	-		+	-	-		7	-	-	-	-	-	-
	3	+		-	-	-		3	-	-	-	-	-	-
4 months after Zika (n = 2)	4	-		-	+	-		122		-	-		-	-
	5	-		-	+	-		146		-	-		-	+
Zika + encephalitis (n = 3)	6	-		+	+	-	6	7	-	-	+	-	+	+
	7	-		+	+	-	6	5	-	-	-	-	-	-
	8	-		+	+	-	5	13	-	-	-	-	-	+
Zika + GBS (n = 7)	9 (2)^4^	-		+	+	BFP^5^	10	10/46^6^	-	-	-	-	-	+
	10 (2)	-		+	+	Classic	33	17/57	-	-	-	-	-	+
	11	-		-	+	Classic	4	6	-	-	-	-	-	-
	12	-	+	+	+	Classic	11	5	-	-	-	-	-	+
	13	-	+	+	+	Classic	10	23	-	-	-	-	-	+
	14	-	+	+	+	MFS^7^	33	5	-	-	-	-	-	+
	15	-		-	+	Classic	3	35	-	-	-	-	-	+
GBS unknown etiology (n = 1)	16	-		-	-	Classic	14	7	-	-	-	-	-	+
Chikungunya (n = 2)	17	-		-	+	Classic	20		-	+	+	-	-	+
	18	-		-	+	Classic	20		-	+	+	-	-	+

^1^Days between onset arboviral symptoms (or fever for patient 16) and neurological onset

^2^Days between onset of arboviral symptoms and sample collection

^3^Plaque reduction neutralization test

^4^Number of samples collected from an individual patient

^5^Bifacial weakness with paresthesias (BFP)

^6^Days between onset of arboviral symptoms and sample collection for first and second samples

^7^Miller Fisher syndrome (MFS)

### Serological analysis

Detection of specific anti-Zika, anti-chikungunya, and anti-dengue IgG antibodies and anti-dengue and anti-chikungunya IgM antibodies were performed using indirect enzyme-linked immunosorbent assays (ELISAs) (Euroimmun, Lüberg, Germany), in accordance with the manufacturer protocol. An IgM antibody-capture ELISA (MAC-ELISA), provided by the Arbovirus Reference Collection division of the Centers for Disease Control and Prevention (CDC), was used in accordance with the established CDC protocol. Detection of RNA for Zika, chikungunya and dengue virus were performed by reverse transcriptase-PCR following published methods [16–18]. Patient samples positive for Zika plaque reduction neutralization test (PRNT) and/or positive for Zika IgM and negative for dengue IgM by ELISA (CDC) were considered positive for Zika infection. Patient 11 was considered positive for Zika infection because it showed positive Zika IgG and negative dengue IgG. Only in patient 15, which is positive for Zika and dengue IgG, lack of cross-reactivity with anti-dengue antibodies could not be confirmed. Zika infection was considered acute when samples were positive for Zika RNA (by RT-PCR, [18] and/or Zika IgM (by ELISA). Biological samples, including blood, were collected upon hospital admission or 4 months after the onset of symptoms, as indicated. Data management was performed using REDCap 6.18.1 - 2018 Vanderbilt University.

### Ganglioside ELISAs

Costar 3700 384-well ELISA plates were coated with single or mixes of Glycolipids (Matreya, Sigma) at 20 μg/ml in 200 proof Molecular Biology ethanol using an Agilent Bravo system in a BSL-2 hood. The lipids used were: sphingomyelin (SPM), phosphatidylserine (PS), sulfatide (SULF), globoside (GS), Trihexosylceramide (CTH-hydroxi fatty acid) (THCH), Trihexosylceramide (CTH non-hydroxy fatty acid)(THCHN), galactocerebroside (GALC), and the gangliosides GM1, GM2, GM3, GA1, GD1A, GD1B, GD2, GD3, GT1B and GQ1B. Plates were then allowed to evaporate at RT after >16 h of incubation at 4°C. Plates were washed 3 times with PBS 0.05% tween 20 and then blocked overnight with PBS supplemented with 3% BSA. Plasma from patients was diluted at 1:100 in blocking buffer and incubated for 2 h at 37 °C. Plates were washed again 3 times and incubated with anti-human IgM/IgG-HRP (Abcam) for 1 h at 37 °C. Plates were washed 3 more times and TMB substrate (BD Biosciences) was added until desired color was obtained. Reaction was stopped with Stop buffer (Biolegend) and absorbance was read at 450 nm. The optical density at 450 nm was compared with the same dilution (1:100) of a positive plasma sample (sample ID: 9b) that was used as reference to calculate relative units (RU). Two negative controls were included in each of the ELISA plates run: (1) The plasma of a healthy US control donor was used in duplicated wells in the ELISA for each glycolipid and combinations. The average of the 2 determinations for each glycolipid and combinations was used as background for each glycolipid and subtracted from each value. (2) The reactivity of each plasma sample in wells coated with PBS supplemented with 3% BSA. The value of eight independent wells for each plasma sample was obtained. It was observed that the variation between the eight replicates with each plasma samples was <0.02 for all samples and the variation between the average values for the different plasma samples was <0.0002. The reactivity of all plasma samples to BSA was considered constant and was not subtracted from assay values. The reactivity to wells coated with only glycolipids coating buffer (ethanol) was not considered since it results in high unspecific background for all samples. The secondary antibody, TMB and stop solution was added using the peristaltic pump on the Biotek EL406. Washes were also done using the 96-head washer on the EL406. Validation of the automated 384-well ELISAs was performed using a similar protocol in 96-well plates with manual pipetting. Five different plasma samples (9b, 10a, 11, 14 and 16) of GBS patients were tested with five randomly chosen glycolipids (SPM, GS, GM1, GD3 and SULF). The variation between the two assays was found to be lower than 0.008 for each of the glycolipids.

### Statistical analysis

Data were analyzed using Prism (GraphPad Software). Unpaired t-test was used to identify statistical differences between groups of samples. For determination of number of antigens recognized per sample and number of positive samples recognizing each ganglioside, reactivity of samples was considered positive if the OD value was at least the average of control background wells plus three times the standard deviation.

## Results

Zika-associated GBS is one of the most serious complications associated with this infection. Our main goal in this study is to compare the prevalence and specificities of anti-ganglioside antibodies in a cohort of Zika-associated GBS patients (n = 7) compared to Zika-infected patients without GBS (n = 8). Among these, some patients presented with encephalitis (n = 3) or were Zika-infected with no neurological symptoms, either in acute phase (n = 3) or 4 months after the onset of symptoms (n = 2). The median of days between the onset of arboviral and neurological symptoms was 10 days for the Zika-associated GBS patients (Table 1).

### Strong anti-ganglioside antibody response in Zika-associated GBS patients

**Fig 1:**
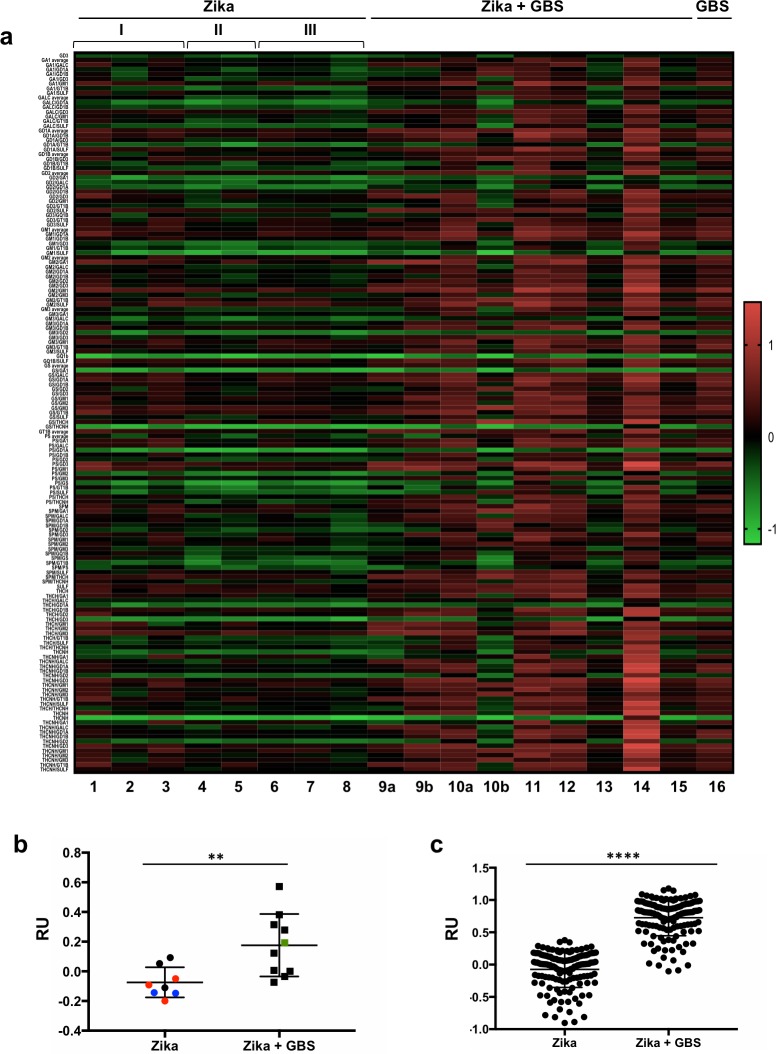
Reactivity of plasma samples from Zika patients with or without GBS. (**a**) Heatmap of antibody reactivity to single lipids and lipid combinations. Results from plasma of patients with uncomplicated Zika (acute (I: patients 1–3) or 4 months after the onset of symptoms (II: patients 4–5), with Zika infection and encephalitis (III: patients 6–8), with Zika infection and GBS (patients 9–15; a and b indicate different time points of sampling for the same patient) or with GBS from unidentified etiology (patient 16). Antigen lipids used: sphingomyelin (SPM), phosphatidylserine (PS), sulfatide (SULF), globoside (GS), Trihexosylceramide (CTH-hydroxi fatty acid) (THCH), Trihexosylceramide (CTH non-hydroxy fatty acid)(THCHN), galactocerebroside (GALC), and the gangliosides GM1, GM2, GM3, GA1, GD1A, GD1B, GD2, GD3, GT1B and GQ1B. (**b**) Average of reactivity values for all lipids and combinations in samples from patients with Zika with or without GBS. Samples of plasma from patients with uncomplicated acute Zika (black circles), 4 months after the onset of symptoms from a Zika infection (blue circles), Zika with encephalitis (red circles) and Zika with GBS (black squares). A sample of plasma from a patient with GBS but no Zika infection (unidentified etiology) (green square) was not considered in the average. (**c**) Average of reactivity values for each lipid and lipid combinations in samples from patients with Zika with or without GBS. Data are expressed as RU values. *p < 0.05, **p < 0.01, ***p < 0.001, ****p < 0.0001, when the groups are compared to each other by unpaired t-test.

Using a novel high throughput ELISA approach, we assessed the plasma of these patients for their reactivity (IgM and IgG) against different gangliosides. The reactivity to combinations of glycolipids has been described to be higher than the reactivity to single ones, possibly due to the formation of complex antigenic structures [12]. We therefore analyzed reactivity against 17 lipids, mostly glycolipids including gangliosides described to be associated with GBS [6], alone or their 139 double combinations. We first performed an overall analysis of all the patient samples to determine the levels of ganglioside reactivity in their plasma using a high throughput ELISA approach to detect anti-ganglioside IgM and IgG antibodies. These assays showed increased anti-ganglioside reactivity in the plasma of Zika-associated GBS patients compared to Zika patients without GBS (Fig 1A–1C). Collectively, these results showed an enriched anti-ganglioside antibody response in the plasma of Zika-associated GBS patients compared to Zika-infected controls.

Additionally, we analyzed one sample from a GBS patient of unknown etiology who was negative for Zika, dengue and chikungunya infections, which showed a similar profile to Zika-associated GBS patients.

**Fig 2:**
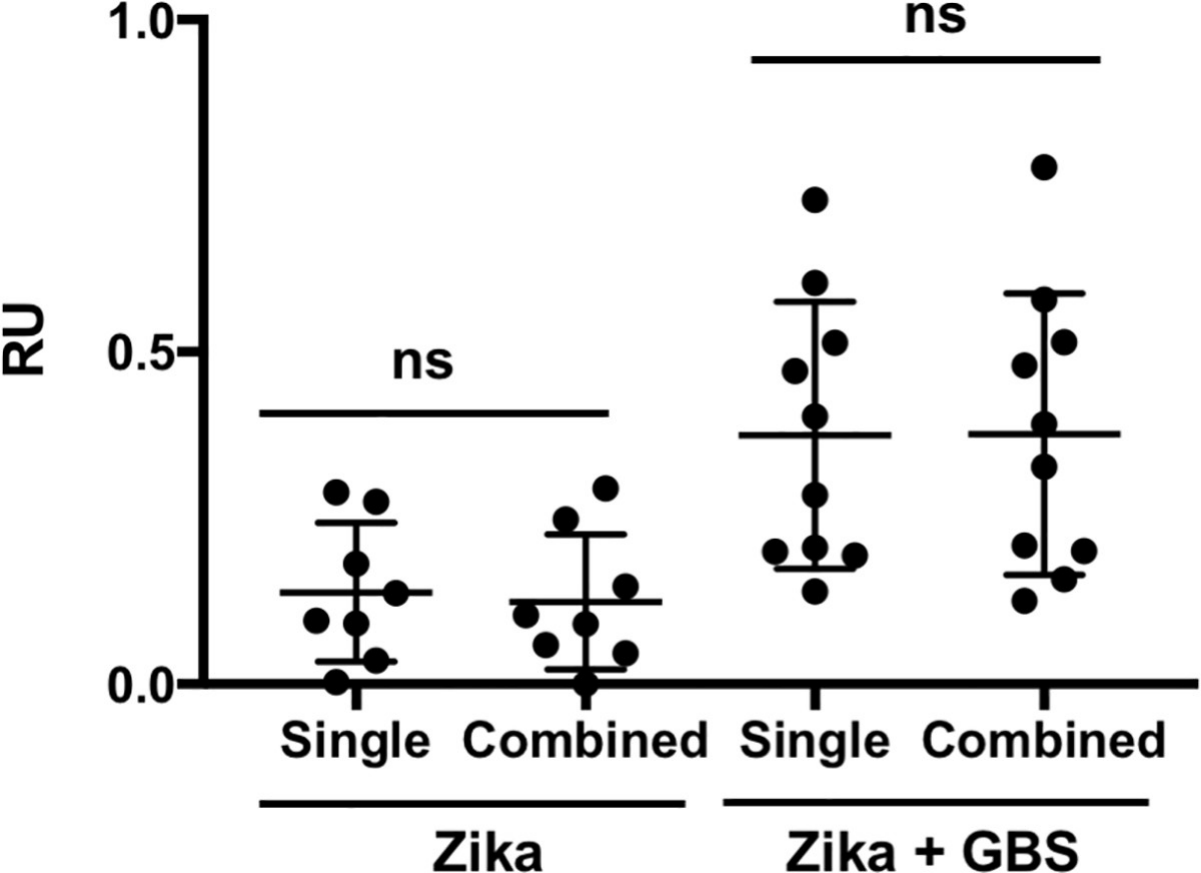
Reactivity to single glycolipids versus combinations. Average antibody reactivity of samples from patients with Zika or Zika + GBS was analyzed for single and combined glycolipids. Each dot represents the average reactivity to all single or combined glycolipids for each plasma sample. No significant (ns) differences between single and combined groups were found when compared by unpaired t-test.

Since a previous report described that the plasma of GBS patients presented higher antibody reactivity to complex glycolipids compared to individual ones, we analyzed the responses to individual versus 2-by-2 combined glycolipids. We did not find any significant differences between the average reactivity of any of the plasma samples to individual or combined glycolipids (Fig 2).

**Fig 3:**
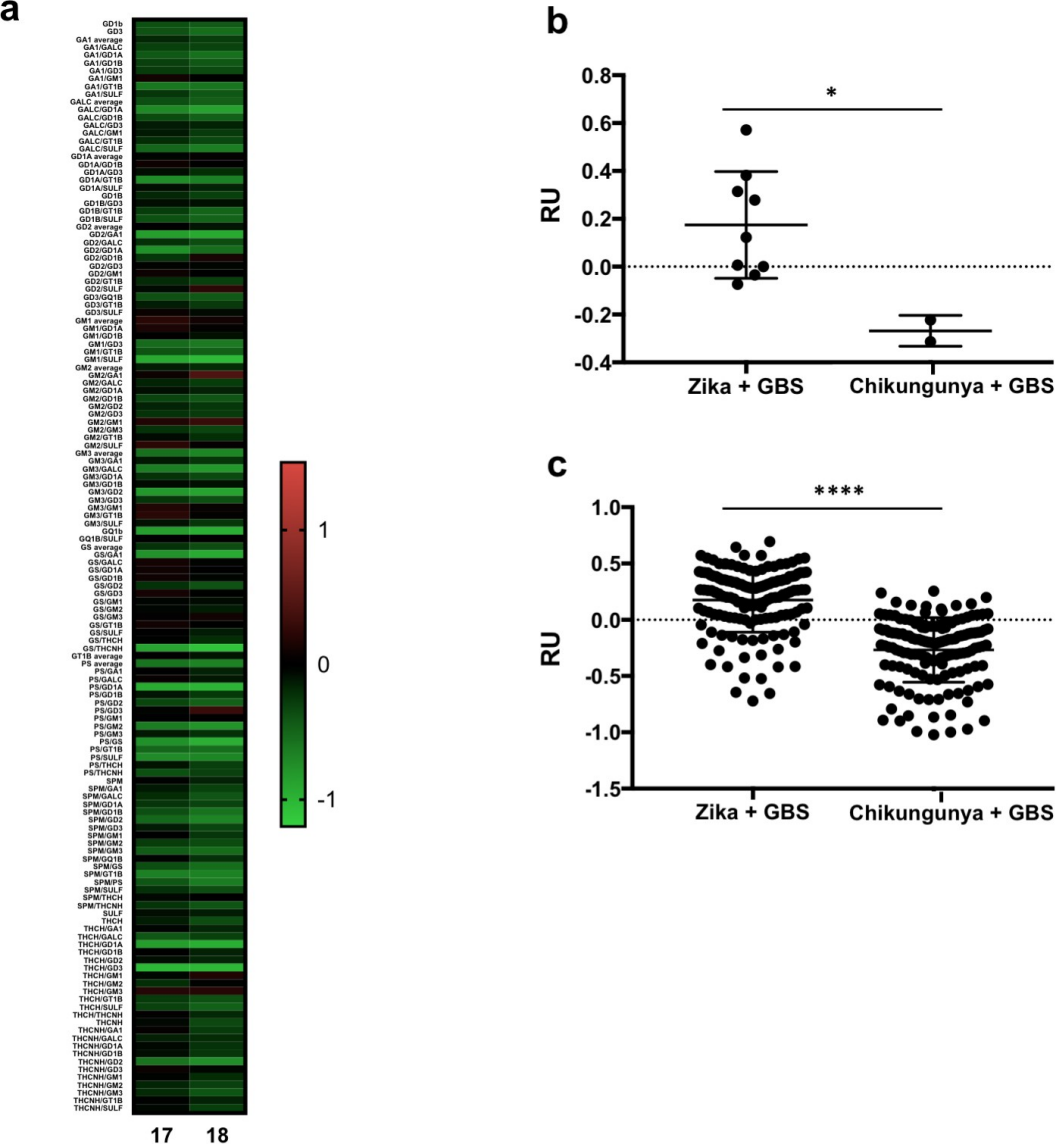
Reactivity of samples from plasma of two GBS patients with active chikungunya virus infection and previous Zika infection (IgG+). (**a**) Heatmap of plasma reactivity to single lipids and lipid combinations. Results from plasma from patients with active chikungunya virus infection (IgM+) and previous Zika infection (IgG+) and GBS compared with Zika infection alone and GBS. (**b**) Average of reactivity values for all lipids and combinations in samples from these patients. (**c**) Average of reactivity values for each lipid and lipid combinations in samples from these patients. Data are expressed as RU values. *p < 0.05, **p < 0.01, ***p < 0.001, ****p < 0.0001, when the groups are compared to each other by unpaired t-test.

We also analyzed the plasma of two GBS patients with active chikungunya virus infection (IgM+) and previous Zika infection (IgG+). The plasma from these patients did not present strong reactivity against gangliosides, in contrast to Zika-associated GBS patients (Fig 3).

### Broad ganglioside specificity in the plasma of Zika-associated GBS patients

**Fig 4:**
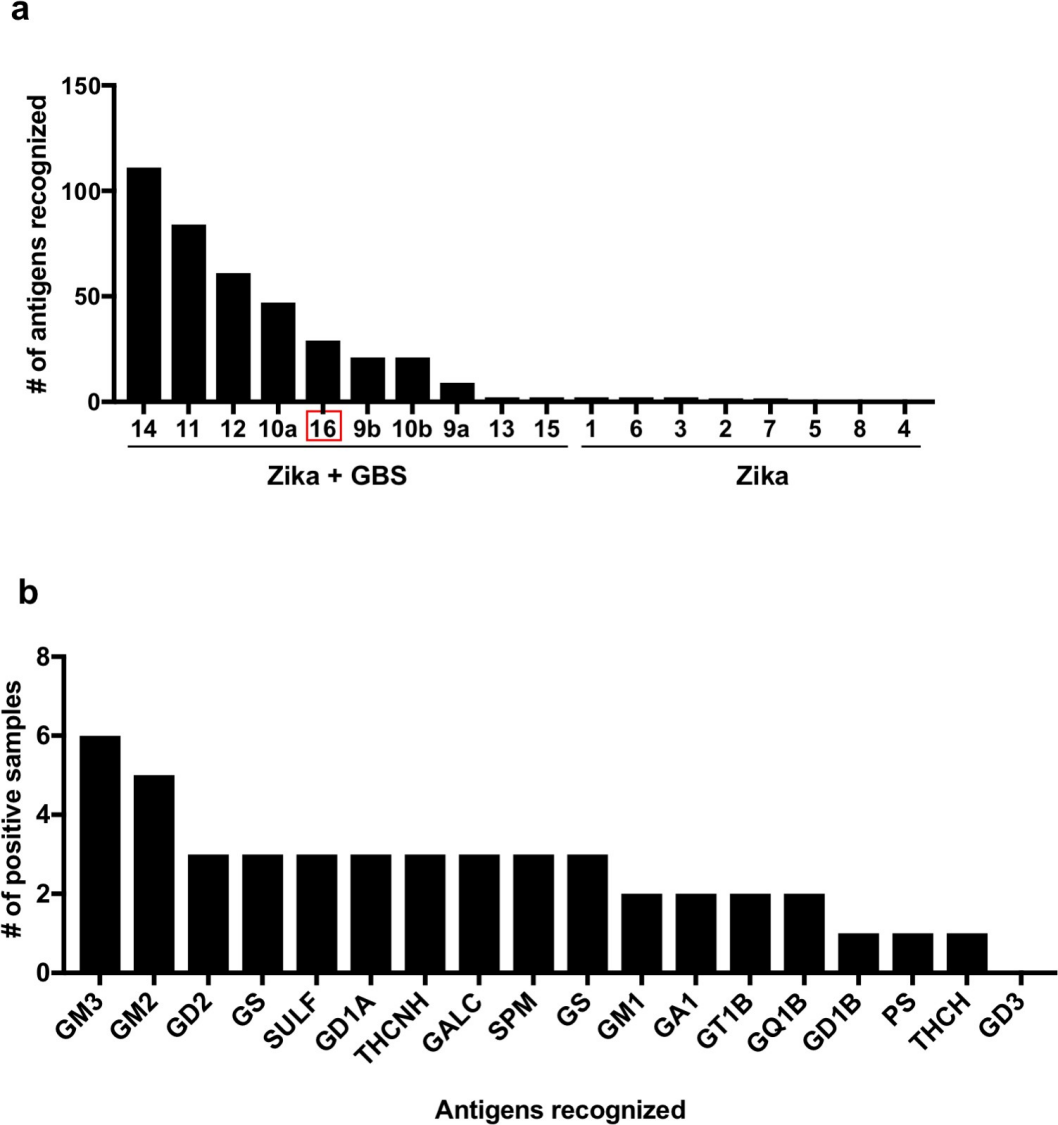
Antigenic analysis of the reactivity of plasma from Zika patients with or without GBS. **(a)** Reactivity to glycolipids, presented single or as combinations, of the plasma samples from Zika patients (identified from 1 to 15; a and b indicate different time points of sampling for the same patient). The patient with GBS of unknown etiology (16) is marked with a red rectangle. Data is presented as number of single lipids or combinations that were recognized by plasma samples of each patient. (b) Reactivity to single glycolipids in plasma samples of Zika-associated GBS patients (n = 9). A sample was considered positive if its value was higher than the average plus 3 three times the standard deviation of the values obtained for the eight Zika without GBS patient samples.

We further dissected the anti-ganglioside reactivity patterns observed in Zika-infected patients with or without GBS. A detailed analysis of antigen reactivity showed that patients that developed GBS recognized a significantly higher number of antigens (single and combined ganglioside mixes) compared to patients without GBS (Fig 4A). The highest number of antigens recognized was found in a GBS patient reacting significantly to >100 different single gangliosides/combinations while all non-GBS patients reacted significantly against 2 or fewer antigens. When we analyzed which specific gangliosides had an enriched reactivity across the Zika infected patients (either in single or combination form) we observed a broad reactivity to different gangliosides and other glycolipids (Fig 4B). These results suggest a broad anti-ganglioside antibody response in Zika-associated GBS patients, independent of combinations.

### Isotypes of antibodies in the anti-ganglioside response in Zika-associated GBS high responders

**Fig 5:**
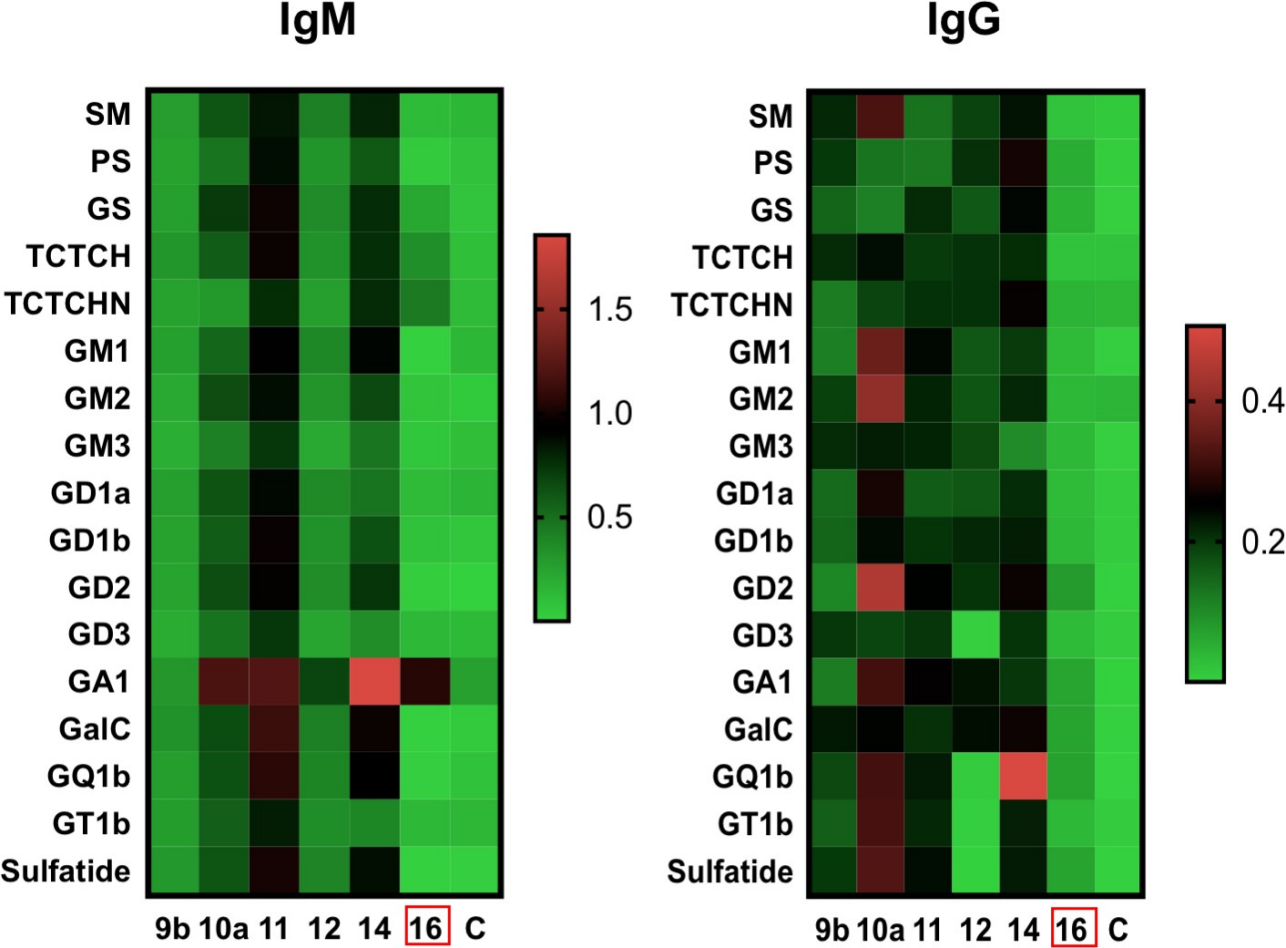
IgM and IgG reactivity of plasma from high-responder Zika patients with GBS. Heatmap of plasma IgM and IgG reactivity to individual glycolipids, expressed as optical density values from the specific ELISAs. The patient with GBS of unknown etiology (16) is marked with a red rectangle. Control uninfected plasma (C).

Our initial screen of anti-ganglioside reactivity evaluated the presence of overall IgM/IgG antibodies in the plasma of the Zika-associated GBS patients. We further analyzed the samples by validating the responses observed in six highly responsive patients, from the Zika-associated GBS group. We also dissected this response by determining IgM and IgG reactivities separately (Fig 5). The results of these assays showed high levels of both anti-ganglioside IgM and IgG in the plasma of the patients compared to control plasma. Additionally, these assays validated the strong broad reactivity of the Zika-GBS patient plasma against a high number of gangliosides (Fig 4). Collectively, these results confirm the presence of IgM and IgG anti-ganglioside antibodies in the plasma of Zika-associated GBS patients. Our findings demonstrate broad reactivity to glycolipids with stronger responses to specific gangliosides.

## Discussion

GBS is one of the most serious complications associated with Zika infection. Neurological symptoms appear shortly after a transient Zika infection resulting in the development of GBS [9, 11, 19, 20]. In this study a median of 10 days before the onset of neurological symptoms was observed, which is similar to a previous report (6 days) [9]. Although Zika virus infection has been reported to lead to development of GBS in patients, little is known about the pathogenesis of this syndrome. Different autoantibodies have been identified as mediators of pathology during different autoimmune disorders, such as anti-nuclear antibodies during Systemic Lupus Erythematosus [21]. Indeed, GBS is considered an autoimmune syndrome due to the immune destruction of peripheral nerve components such as gangliosides [19, 22, 23]. Generation of anti-ganglioside antibodies and other autoantibodies has been reported in non-Zika infection-induced GBS, such as the classical one induced by the bacterium *C*. *jejuni* [7]. Because anti-ganglioside autoantibodies are implicated in the pathogenesis of GBS [6], we sought to determine whether anti-ganglioside antibodies were selectively increased in Zika-infected patients with GBS as opposed to Zika-infected controls with self-limited illness.

Our initials results show a potent broad reactivity against single and combination of 17 different gangliosides compared to non-GBS Zika infected patients. These anti-ganglioside antibodies were both of the IgM and IgG isotypes. Both anti-ganglioside IgG and IgM have been suggested to have a pathological role during different non-Zika infection induced GBS patients [24]. The mechanism by which these anti-gangliosides lead to pathology is poorly understood. The significant increase in anti-ganglioside IgM/IgG antibodies in patients with GBS compared to non-GBS Zika-infected patients suggests a role for these antibodies in mediating the disease.

A recent study assessed a similar relationship of ganglioside reactivity in Zika-associated GBS patients in French Polynesia [9]. This study found an increase in general anti-ganglioside reactivity against a different set of gangliosides tested by a different method of combinatorial glycolipid microarray. Our results show reactivity against different individual and combination of gangliosides hence illustrating the diversity in the anti-ganglioside response induced in a different cohort of Zika-associated GBS patients. We did not observe any enhancement of reactivity whenever specific gangliosides were tested individually or in combination, which may be attributed to the different methods used for detection (microarray versus ELISA).

In addition to Zika virus, other arbovirus infections like dengue and chikungunya have also been reported to lead to autoimmunity and neurological problems such as GBS [25–30]. A large percentage of the patients in our study had previous, but not active, dengue infections, as indicated by the differential anti-dengue IgM and IgG reactivity. Previous dengue infections are expected in this area of Brazil where prevalence is 86% in adults and where preexisting high antibody titers to dengue virus have been associated with reduced risk of Zika infection [31].

We also assessed the plasma of two GBS patients with active chikungunya virus infection and previous Zika infection (IgG+). When we assessed the plasma of these patients for anti-ganglioside reactivity, our results demonstrated levels of anti-ganglioside antibodies significantly lower than GBS patients with an active Zika infection. It is possible that anti-ganglioside antibodies in the circulation induced during a Zika infection decrease over time and are no longer present in these patients. The role of an active chikungunya infection is unclear.

It is well established that Zika can infect neurons [32–34], including peripheral motor nerves/nerve roots, which have high abundance of different gangliosides. Direct infection of neurons would target these cells for phagocytosis by antigen presenting cells, enabling presentation of many auto antigens, such as gangliosides, along with virus antigens, resulting in an antibody response against both. Increased immune recognition of virus-infected neuronal cell antigens could be at the basis of Zika–induced GBS. Immune response against direct neural infection would be consistent with the observation that GBS tends to be an early complication of Zika. Accordingly, a recent studied showed how antiviral CD8+ T-cells mediated nerve damage leading to paralysis in Zika-infected mice [35]. Additionally, immune mediated neurological damage was also reported in fatal cases of Zika-induced microcephaly [36], providing additional evidence of an immune component contributing to the neuronal damage leading to GBS. Accordingly, plasma from Brazilian Zika-infected patients recognized GD3 from neurons in retina tissues [10]. These patients were found to have high titers of anti-ganglioside antibodies, mainly anti-GD3 IgG antibodies. Nevertheless, mechanistic studies are needed to test this hypothesis and elucidate the role anti-ganglioside antibodies might have in Zika-induced GBS.

Although we observed wide reactivity to gangliosides, our results also showed differential reactivity to some gangliosides in the plasma of Zika-associated GBS patients. When we dissected these responses by validating them with single ganglioside ELISAs, we confirmed a strong reactivity against specific gangliosides such as GA1. Interestingly, GA1 also had the highest reactivity from the French Polynesia study as assessed by a different method of combinatorial glycolipid microarray [9]. However, in a different study in India with Zika-infected GBS patients, the most commonly recognize ganglioside was GT1b [37]. Collectively, our results suggest that, in a minor subset of infected patients, Zika infection causes neuronal damage that triggers an auto-immune antibody response against neuron-derived ganglioside antigens, which contributes to the pathogenesis of GBS.
